# Disseminated *Mycobacterium genavense* presenting as recurrent steroid-responsive mesenteric lymphadenitis

**DOI:** 10.1128/asmcr.00067-26

**Published:** 2026-07-29

**Authors:** Portia Mira, Nancy L. Wengenack, Mary J. Kasten

**Affiliations:** 1 Department of Laboratory Medicine and Pathology, Mayo Clinic 6915, Rochester, Minnesota, USA; 2 Department of Medicine, Section of Infectious Diseases, Mayo Clinic 6915, Rochester, Minnesota, USA; Pattern Bioscience, Austin, Texas, USA

**Keywords:** *Mycobacterium genavense*, non-tuberculous mycobacteria, lupus, mesenteric lymphadenitis, cell-free DNA

## Abstract

**Background:**

*Mycobacterium genavense* is a rare opportunistic non-tuberculous mycobacterium primarily reported in advanced human immunodeficiency virus infection.

**Case Summary:**

A 70-year-old woman with systemic lupus erythematosus developed progressive abdominal pain, weight loss, and mesenteric lymphadenitis repeatedly responsive to corticosteroids. Endoscopic biopsies revealed abundant acid-fast bacilli, and molecular testing confirmed the presence of *M. genavense*. Treatment with azithromycin, ethambutol, and rifabutin led to gradual recovery.

**Conclusion:**

Steroid-responsive mesenteric inflammation in immunocompromised patients should prompt evaluation for infection, including mycobacteria and other culture-negative microorganisms using molecular diagnostics. This case report highlights the importance of plasma microbial cell-free DNA assays in the identification of fastidious organisms, like *M. genavense*, and, in some cases, serial testing may be useful to monitor treatment response.

## INTRODUCTION

Non-tuberculous mycobacteria are increasingly recognized causes of opportunistic infection in immunocompromised hosts, with expanding clinical relevance beyond traditional populations such as individuals with advanced human immunodeficiency virus (HIV) infection (1–3). *Mycobacterium genavense* is a rare, fastidious species that presents unique diagnostic challenges due to its slow growth, requirement for supplementation to permit growth, frequent culture negativity, and requirement for specialized molecular methods for identification (4–6). Historically, disseminated *M. genavense* infection has been described predominantly in patients with profound cellular immunodeficiency, particularly those with acquired immunodeficiency syndrome (7, 8).

In recent years, however, cases have been increasingly reported in non-HIV immunocompromised patients, including solid organ transplant recipients and individuals receiving long-term immunosuppressive therapy for autoimmune or inflammatory disease (9, 10). Clinical manifestations are often non-specific and may include constitutional symptoms, abdominal pain, diarrhea, weight loss, lymphadenopathy, and elevated inflammatory markers (5, 8). Gastrointestinal (GI) involvement, while well documented in disseminated disease, remains an uncommon presentation and is frequently mistaken for inflammatory bowel disease, malignancy, or autoimmune-mediated inflammation (6, 10).

Mistaking *M. genavense* infection for an autoimmune disease or an immune-mediated inflammatory disease may delay definitive evaluation for infectious etiologies and permit ongoing mycobacterial proliferation under conditions of intense immunosuppression (6, 11, 12). Patients may temporarily respond to steroids, falsely reinforcing a presumed autoimmune diagnosis, delaying definitive evaluation for infectious etiologies, potentially resulting in disseminated infection and severe disease (6, 11, 12).

The advent of molecular diagnostics, including broad-range polymerase chain reaction combined with sequencing and plasma microbial cell-free DNA (mcfDNA) sequencing, has substantially improved the ability to identify fastidious pathogens such as *M. genavense* when conventional cultures are unrevealing. These tools also offer potential utility in disease monitoring and assessment of treatment response, though data remain limited (13, 14).

We report a case of disseminated gastrointestinal *M. genavense* infection in a patient with systemic lupus erythematosus (SLE) receiving chronic immunosuppression, whose clinical course was marked by recurrent steroid-responsive mesenteric lymphadenitis, delayed diagnosis, and eventual identification through histopathology and molecular testing. This case illustrates anchoring bias and paradoxical response to steroids—two critical diagnostic pitfalls with infection. The case underscores the importance of maintaining suspicion for mycobacterial infection in unexplained or steroid-responsive inflammation in immunocompromised patients and highlights the critical role of tissue diagnosis and the usefulness of molecular testing with fastidious organisms like *M. genavense*.

## CASE PRESENTATION

**Fig 1:**
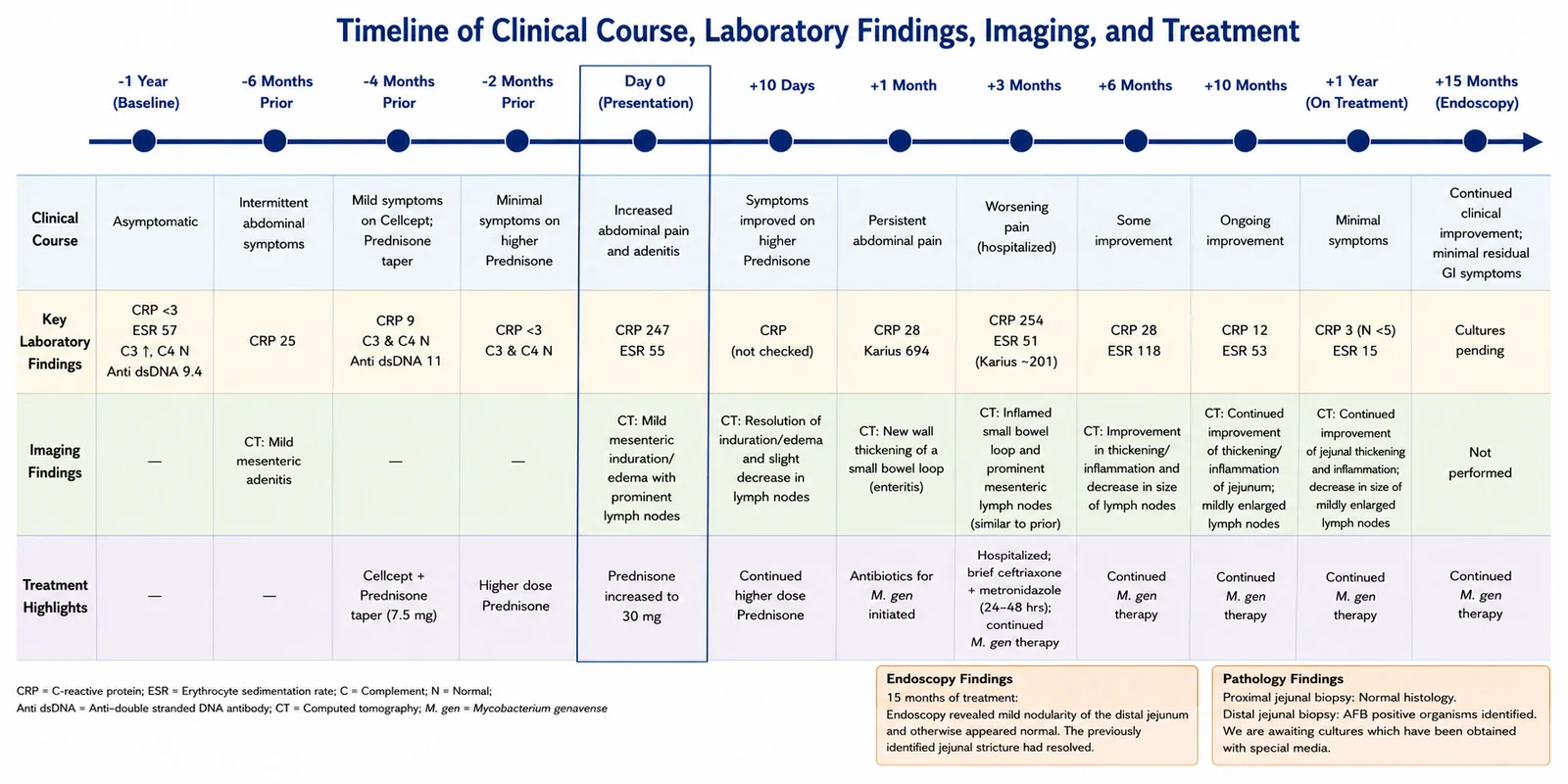
Clinical timeline demonstrating the patient’s disease course, inflammatory markers, imaging findings, and treatment response during disseminated gastrointestinal *Mycobacterium genavense* (*M. gen*) infection.

A 70-year-old retired nurse with a long-standing history of SLE with a remote history of glomerular nephritis, rheumatoid arthritis, hypertension, and a history of stroke related to antiphospholipid antibody syndrome subsequently on chronic warfarin presented with increased abdominal pain on top of low-grade chronic abdominal pain and anorexia 8 months prior to her infectious disease evaluation. The patient’s weight decreased from 62 kg to 52 kg over the past year, with most of the weight loss being over the preceding 4 months. She was found approximately 8 months prior to her infectious disease evaluation to have a mildly elevated CRP of 25 mg/L (reference value <5.0 mg/L) and new indeterminate mid-mesenteric lymph nodes with fat stranding. No inflammation of her small bowel or colon was seen on a CT of the abdomen and pelvis. She was given a dose of solumedrol followed by an increase in her 3 mg dose of chronic prednisone to 30 mg, which was tapered over 10 days to 5 mg. Her abdominal pain resolved, and CRP returned to normal (Fig. 1).

Unfortunately, pain recurred, and her prednisone was increased to 10 mg with improvement. She was therefore initiated on azathioprine 8 weeks later, with the likelihood that the mesenteric lymphadenitis was autoimmune in nature and related to SLE. Azathioprine caused diarrhea, and she was changed to mycophenolate mofetil with a steroid taper. Her pain improved but then mildly recurred, associated with anorexia and nausea after 10 weeks of mycophenolate mofetil. CT of her abdomen was essentially normal, with resolution of the previously seen lymphadenitis. Six months following her original presentation, her pain significantly increased, and CRP increased to 247 mg/L (reference value <5.0 mg/L). A repeat CT of the abdomen again revealed mild mesenteric induration or edema with mildly increased lymph nodes (Fig. 1). Her prednisone was increased to 20 mg daily, and mycophenolate was discontinued. A repeat CT of the abdomen 10 days later revealed resolution of the mesenteric inflammation. She was treated with a 21-day course of amoxicillin/clavulanic acid 875/125 mg by mouth twice daily for possible small bowel overgrowth without improvement. She also reported intermittent low-grade fever and drenching sweats. She was seen by gastroenterology and was found to have erythematous mucosa in the gastric body and antrum on endoscopy. AFB stain of her small bowel demonstrated numerous mycobacteria within histiocytic aggregates. A repeat biopsy when the patient was off antibiotics again demonstrated numerous mycobacteria in her small bowel. Tissue from her duodenal biopsy was sent for 16S broad-range bacterial PCR with sequencing testing and was positive for *M. genavense*. Additionally, microbial cell-free DNA testing of her plasma, via Karius (13), was positive for *M. genavense* at 694 molecules per 100 nL and was the only organism reported. Mycobacterial cultures of duodenal aspirates and of tissue from her small bowel were negative (Fig. 1).

The patient was seen in the infectious disease clinic and started on azithromycin 250 mg, ethambutol 800 mg, and rifabutin 300 mg by mouth once daily (BMI 18.9). Unfortunately, after 3 weeks of treatment, her pain significantly increased and was localized to her left lower quadrant. She also had severe nausea and vomiting aggravated by eating. CT demonstrated an inflamed small bowel in the left lower quadrant. Her CRP, which had nearly normalized, was again severely elevated to 258 mg/L (reference value <5.0 mg/L). She was hospitalized for fluids; her antimycobacterial treatment was temporarily discontinued for 48 hours, and she was treated with ceftriaxone IV 2 g q 24 and metronidazole 500 mg q8 for about 36 hours. A colonoscopy was unrevealing, but a capsule endoscopy revealed numerous white plaques or nodules with mildly erythematous rims starting in the jejunum and continuing distally. A repeat biopsy again revealed numerous AFB within macrophages. She improved with the colonoscopy prep and ondansetron. Her gastroenterologist believes she had a partial small bowel obstruction that was relieved with the colonoscopy prep. Fifteen months later, she remained on three drugs for treatment with minimal abdominal pain and very gradual improvement of her weight up to 61 kg (BMI 23).

The initial plan was to treat for at least 1 year. After 15 months of treatment, a repeat capsule endoscopy revealed mild nodularity of her distal jejunum, but otherwise, her bowel appeared normal. Biopsy of her proximal jejunum was normal, and the distal jejunum revealed Kinyoun stain positive for AFB. At the time of this case report being submitted, there have not yet been any cultures positive for *M. genavense*, despite the use of media with Mycobactin J supplementation (Fig. 1).

## DISCUSSION

*Mycobacterium genavense* is a rare, fastidious non-tuberculous *Mycobacterium* that has historically been associated with disseminated infection in patients with advanced HIV infection (2, 6). It has been noted to have tropism for the GI tract and frequently presents with GI symptoms (15). Unlike many other non-tuberculous mycobacteria, *M. genavense* is notoriously difficult to culture using standard laboratory techniques, often requiring prolonged incubation, specialized media supplemented with Mycobactin J, or remaining culture negative altogether (6). In this case, the original mycobacterial culture was negative, likely due to failure to supplement with Mycobactin J because *M. genavense* was not fully appreciated as a potential pathogen at the time. As a result, diagnosis frequently relies on histopathologic findings and molecular methods, contributing to delays in recognition and treatment.

Although initially described in profoundly immunocompromised people living with HIV, *M. genavense* infection has increasingly been reported in non-HIV populations, including solid organ transplant recipients and patients undergoing chronic immunosuppressive therapy for autoimmune or inflammatory conditions (7, 8). Corticosteroids, antimetabolites, and other immunomodulatory agents impair cell-mediated immunity, creating a permissive environment for intracellular pathogens such as *M. genavense*. In this context, the patient’s long-standing systemic lupus erythematosus, cumulative corticosteroid exposure, and additional immunosuppressive therapies likely contributed substantially to disease susceptibility and severity of illness.

This case illustrates the diagnostic challenges of *M. genavense* and similar fastidious infections presenting as steroid-responsive mesenteric inflammation in immunocompromised patients, highlighting the importance of maintaining a high index of suspicion for infection. Although plasma mcfDNA sequencing offers an appealing, non-invasive means of monitoring pathogen burden over time, its role in assessing treatment response remains insufficiently validated. Conceptually, serial quantification of circulating microbial DNA could provide an objective marker of microbial clearance, particularly in infections where cultures are repeatedly negative or difficult to obtain, as in *M. genavense*. However, real-world data highlight important limitations. A 2026 retrospective analysis of more than 170 episodes of serial plasma mcfDNA testing found that over 95% of serial tests did not meaningfully affect clinical management (16), and the authors noted the absence of published evidence establishing mcfDNA kinetics as a reliable tool for tracking therapeutic response. This contrasts with traditional monitoring strategies such as serial blood cultures or quantitative viral loads, where established kinetic patterns inform clinical decision-making. Some studies in highly immunocompromised populations suggest that serial mcfDNA holds promise for pathogen surveillance, particularly for viruses (17, 18): in hematopoietic stem-cell transplant recipients, serial Karius testing demonstrated strong correlation with CMV quantitative PCR and often detected pathogens earlier than standard microbiologic testing (19). A limitation of our work is that, as a single-patient case report, the findings may not be generalizable to broader immunocompromised populations or predict treatment outcomes in other patients with disseminated *M. genavense* infections. Taken together, current evidence underscores significant interest but limited proof supporting the use of serial plasma mcfDNA assays for treatment response monitoring.

Our case highlights the diagnostic complexity of disseminated *Mycobacterium genavense* infection in a non-HIV immunocompromised patient, particularly when clinical and radiographic findings mimic autoimmune disease. Recurrent steroid-responsive mesenteric lymphadenitis, constitutional symptoms, and progressive gastrointestinal involvement delayed recognition of an underlying opportunistic infection and reinforced presumptive autoimmune inflammation in this patient. The eventual diagnosis required integration of clinical reasoning, histopathology, molecular testing of tissue, and plasma microbial cell-free DNA sequencing due to the organism’s fastidious nature and limited recoverability by conventional culture methods. Our case underscores the importance of maintaining a broad infectious disease differential in immunosuppressed patients with persistent or recurrent inflammatory findings, even when symptoms mimic autoimmune disease and initially improve with corticosteroids. Early tissue sampling and incorporation of advanced molecular diagnostics may facilitate more timely diagnosis and treatment of rare non-tuberculous mycobacterial infections.
